# Coexisting *RET/PTC* and *TERT* Promoter Mutation Predict Poor Prognosis but Effective RET and MEK Targeting in Thyroid Cancer

**DOI:** 10.1210/clinem/dgae327

**Published:** 2024-05-13

**Authors:** Wei Zhang, Shuhuang Lin, Zhuo Wang, Wenyong Zhang, Mingzhao Xing

**Affiliations:** Thyroid Research Institute, School of Medicine, Southern University of Science and Technology, Shenzhen, Guangdong 518055, PR China; Thyroid Research Institute, School of Medicine, Southern University of Science and Technology, Shenzhen, Guangdong 518055, PR China; Thyroid Research Institute, School of Medicine, Southern University of Science and Technology, Shenzhen, Guangdong 518055, PR China; Thyroid Research Institute, School of Medicine, Southern University of Science and Technology, Shenzhen, Guangdong 518055, PR China; Thyroid Research Institute, School of Medicine, Southern University of Science and Technology, Shenzhen, Guangdong 518055, PR China

**Keywords:** *RET/PTC*, *TERT* promoter mutation, thyroid cancer, mortality, prognostic biomarker

## Abstract

**Context:**

The role of *RET/PTC* rearrangement in the clinical outcomes of papillary thyroid cancer (PTC) is controversial and remains to be clearly undefined.

**Objective:**

This work aimed to investigate the role of coexisting *RET/PTC* rearrangement and *TERT* promoter mutation in the prognosis and therapeutic targeting in PTC.

**Methods:**

A total of 669 PTC patients with complete clinical follow-up and genetic data were pooled from thyroid cancer data sets TCGA-THCA, MSK-MetTropism, and MSK-IMPACT, from whom 163 patients (112 women and 47 men, 4 unknown) with wild-type (WT) *BRAF/RAS* were identified, with a median age (interquartile range [IQR]) of 46.00 (33.00-61.00) years and a median follow-up time (IQR) of 16.13 (8.09-27.91) months for comparative genotype cohort analysis of mortality.

**Results:**

There was a significant concurrence index between *RET/PTC* and *TERT* promoter mutations, being 2.040 (95% CI, 1.110-3.747; *P* = .023). Mortality occurred in 5 of 100 (5%) patients harboring neither mutation, 2 of 18 (11.1%) patients harboring a *TERT* promoter mutation alone, 0 of 31 (0%) patients harboring a *RET/PTC* alone, and 7 of 14 (50%) patients harboring both genetic alterations, corresponding to hazard ratios (95% CI) of 1 (reference), 2.469 (0.405-14.022), 3.296e-09 (0-inf), and 9.019 (2.635-30.870), respectively, which remained essentially unchanged after adjustment for patient race, sex, and age. Similar results were observed with *BRAF/RAS* and *TERT* promoter mutations. Mechanistically, RET/PTC used the MAP kinase pathway to upregulate the mutated *TERT*, but not the WT *TERT*, and, correspondingly, targeting RET and MEK could suppress mutated *TERT* but not the WT *TERT*.

**Conclusion:**

Coexisting *RET/PTC* and *TERT* promoter mutation identify PTC as a unique clinical entity with high mortality, providing new implications for genetic-based prognostication and potential therapeutic targeting of RET and MEK guided by *RET*/*PTC* and *TERT* status.

Thyroid cancer is a common endocrine malignancy, with papillary thyroid cancer (PTC) being the most common type, accounting for up to more than 90% of all thyroid malignancies (1-3). The common driver genetic alterations of PTC include *BRAF* mutations, *RAS* mutations and *RET/PTC* rearrangements in the mitogen-activated protein kinase kinase (MAPK) pathway and telomerase reverse transcriptase (*TERT*) gene promoter mutations (4-6). The most common *TERT* promoter mutations in PTC are Chr.5:1 295 228 C > T (termed *TERT* C228T) and Chr.5:1 295 250 C > T (termed *TERT* C250T), which form the binding sites for ETS transcriptional factors, driving the expression of *TERT* (7). *BRAF*/*RAS* mutations and *TERT* promoter mutations commonly coexist to form genetic duets that drive poor clinical outcomes of PTC and hence are the main players in current genetic-based risk management of PTC (8-18) and some other cancers as well, such as melanoma (19-21). An important underlying molecular mechanism is that the mutant BRAF/RAS-activated MAPK pathway, through various downstream signaling routes/molecules, selectively activates the mutated *TERT* promoter, not the wild-type (WT) *TERT*, resulting in robust *TERT* expression and TERT-induced oncogenic consequences, particularly apoptosis resistance (22-27).

RET is a transmembrane glycoprotein receptor tyrosine kinase, encoded by the proto-oncogene *RET* and containing an intracellular catalytic tyrosine kinase domain (28-30). Activating genetic alterations of *RET* include mutations in familial medullary thyroid cancer and MEN2 syndrome (31-33) and, more commonly, genetic rearrangements in sporadic PTC—seen in about 25% of adult PTC and 50% of pediatric PTC (4, 5, 28-30).

*RET* rearrangement was first identified in PTC, hence designated as *RET/PTC*, in which the RET tyrosine kinase domain is fused to the N-terminus of a partner gene, resulting in ligand-independent constitutive kinase activation (34), which can activate both MAPK and PI3K pathways, causing oncogenesis and tumor progression (35). There are many types of *RET/PTC* rearrangements defined by different fusion partner genes, among which *RET/PTC1* and *RET/PTC3* are the most common. Unlike *BRAF/RAS* mutations, the clinical behaviors and oncogenic nature of *RET/PTC* rearrangements in PTC are controversial, although they are often shown to be nonaggressive on general analysis (4, 5, 28-30). This generates a dilemma in their clinical prognostic application. Inspired by the coexisting *BRAF/RAS* mutations and *TERT* promoter mutations in synergistically driving the aggressiveness of PTC, we hypothesize that the *TERT* promoter status may determine the nature of *RET/PTC* in affecting clinical outcomes. We tested this hypothesis by analyzing the effects of these genetic alterations on the mortality of PTC patients and experimentally defining the molecular mechanism.

## Materials and Methods

### Clinical Cohorts and Mutational Status

This study used 3 thyroid cancer data sets—TCGA-THCA (36), MSK-MetTropism (37), and MSK-IMPACT (38). Clinical and genomic mutation data were obtained from the cBioPortal for Cancer Genomics (https://www.cbioportal.org), which are summarized in Supplementary Table S1 in the supplementary materials (39). A total of 669 PTC patients with complete clinical follow-up and genetic data were pooled from the 3 thyroid cancer data sets (Supplementary Table S2 in the Supplementary Materials (39)), from whom 163 patients (112 women and 47 men, 4 unknown sex) harboring WT *BRAF/RAS* were identified, with a median age (interquartile range [IQR]) of 46.00 (33.00-61.00) years and a median clinical follow-up time (IQR) of 16.13 (8.09-27.91) months. A positive mutation was designated when the PTC sample exhibited genetic changes annotated in ClinVar (https://www.ncbi.nlm.nih.gov/clinvar) or it appeared in the 3 thyroid cancer data sets (36-38). Ten *RET/PTC* rearrangements were identified, including *RET* fusions with *CCDC6*, *NCOA4*, *TIMM23B*, *ERC1*, *AKAP13*, *BMS1*, *FKBP15*, *TRIM24*, *RUFY3*, and *SPECC1L. TERT* promoter mutations included *TERT* C228T, *TERT* C250T, and *TERT* C242T. *BRAF* mutations included *BRAF* V600E, *BRAF* G469A, and *BRAF* fusions with *AKAP9*, *EXOC4*, and *SND1*. *RAS* mutations included G13R, Q61K, and Q61R in *KRAS*, *HRAS*, and *NRAS* genes.

### Cohort Comparative Analysis of Patient Mortality and Survival

Given the well-established association between *BRAF/RAS* mutations and *TERT* promoter mutations as well as their synergistic oncogenic effects on the aggressiveness of PTC, our comparative analysis focused mainly on individuals with WT *BRAF/RAS* to avoid the influence from mutations in these genes and studied purely the genetic role of *RET/PTC* and *TERT*. For comparative cohort analyses of the effects of *RET/PTC* rearrangements and *TERT* promoter mutations on clinical outcomes, namely the overall mortality and survival of patients, this cohort with WT *BRAF*/*RAS* was divided into various genotype groups as defined in “Results.” The survival intervals were defined as the period from the time of PTC diagnosis to the patient's death or the time of their last follow-up if alive.

### In vitro Study of the Role of *RET/PTC* in the Regulation of *TERT*

TPC-1 cells harboring *RET/PTC1* and *TERT* C228T and LC2/AD cells harboring *RET/PTC1* and WT *TERT* were used to investigate the molecular mechanism underlying the role of RET/PTC in the regulation of *TERT* and hence its role in clinical outcomes of PTC. Standard cellular and molecular approaches, including cell culture, treatments with RET/PTC inhibitor LOXO292 (selpercatinib) and MEK inhibitor AZD6244 (selumetinib), RNA isolation and real-time quantitative polymerase chain reaction, Western blotting, dual-luciferase reporter assay for the *hTERT* promoter, and CRISPR/Cas9 editing are detailed in the supplementary materials (39).

### Statistical Analysis

Categorical data were summarized as frequencies and percentages. Continuous data were summarized as medians and IQR. The *χ*^2^ test was used to analyze categorical variables and Wilcoxon-Mann-Whitney test to analyze continuous variables. The life-table method was used to determine cumulative mortality and log-rank test to construct Kaplan-Meier survival curves. Cox proportional hazard regression analysis was used to examine hazard ratios (HRs) for the effects of genetic alterations on mortality. All *P* values were 2-tailed and a *P* less than .05 was considered statistically significant. Statistical analyses were performed using R (version 4.2.3) and its appropriate packages (40). Paired *t* test was used to compare gene expression levels. *TERT* promoter activities were compared using unpaired *t* test.

## Results

### Conversion of *RET/PTC* by Coexisting *TERT* Promoter Mutation Into a Strong Driver in the Aggressiveness of Papillary Thyroid Cancer

Consistent with the current knowledge, the overall analysis of the pooled data from the 3 data sets showed mutual exclusion among *BRAF* mutations, *RAS* mutations, and *RET/PTC* rearrangements in the MAPK pathway and coexistence of *BRAF/RAS* mutations with *TERT* promoter mutations (Supplementary Fig. S1A in the supplementary materials (39)). The most common *RET/PTC* rearrangements were with partner fusion genes *CCDC6* and *NCOA4* to form *RET/PTC1* and *RET/PTC3*, respectively (Supplementary Fig. S1B in the supplementary materials (39)). From the 3 data sets, we identified 669 individuals with complete clinical follow-up and genetic data and their demographic characteristics are summarized in Supplementary Table S2 in the supplementary materials (39). As genetic duets of coexisting *BRAF/RAS* mutations and *TERT* promoter mutations are well known to be associated with aggressiveness of PTC, we identified a cohort of 163 patients harboring WT *BRAF/RAS* to study the role of *RET/PTC* and *TERT* and their demographic characteristics with respect to the *RET/PTC* and *TERT* status, which are summarized in Table 1. The genetic duet of concurrent *RET/PTC* and *TERT* promoter mutations was seen in 14 of 163 (8.59%) cases in this cohort. Concurrence test of the interactions between *RET/PTC* rearrangements and *TERT* promoter mutations revealed a concurrence index of 2.040 (95% CI, 1.110-3.747; *P* = .023) (Table 2), demonstrating a significant association between them.

**Table 1.** Demographic and genetic characteristics of the patient cohort with wild-type *BRAF/RAS*

Clinicopathological characteristics		Overall (N = 163)	*RET/PTC* Wt*^a^*		*RET/PTC* rearrangement*^b^*	
			*TERT*p Wt*^c^* (N = 100)	*TERT*p Mt*^d^* (N = 18)	*TERT*p Wt*^c^* (N = 31)	*TERT*p Mt*^d^* (14)
Age, y	Median (IQR)	46.00 (33.00-61.00)	46.00 (33.01-61.00)	32.00 (27.00-46.00)	61.53 (42.40-65.07)	69.00 (60.62-74.59)
	Missing Info, No. (%)	12 (7.4)	7 (7.0)	0 (0)	2 (6.5)	3 (21.4)
Sex	Female, No. (%)	112 (68.7)	73 (73.0)	7 (38.9)	22 (71.0)	10 (71.4)
	Male, No. (%)	47 (28.8)	23 (23.0)	11 (61.1)	9 (29.0)	4 (28.6)
	Missing Info, No. (%)	4 (2.5)	4 (4.0)	0 (0)	0 (0)	0 (0)
Follow-up time, mo	Median (IQR)	16.13 (8.09-27.91)	14.80 (7.16-28.46)	13.57 (3.38-24.18)	23.84 (11.54-27.49)	17.74 (15.42-31.19)
Race	White, No. (%)	91 (55.8)	51 (51.0)	13 (72.2)	19 (61.3)	8 (57.1)
	Asian, No. (%)	21 (12.9)	10 (10.0)	3 (16.7)	7 (22.6)	1 (7.1)
	Black, No. (%)	10 (6.1)	8 (8.0)	1 (5.6)	0 (0)	1 (7.1)
	Other, No. (%)	41 (25.2)	31 (31.0)	1 (5.6)	5 (16.1)	4 (28.6)
Death	Yes, No. (%)	14 (8.6)	5 (5.0)	2 (11.1)	0 (0)	7 (50)
	No, No. (%)	149 (91.4)	95 (95.0)	16 (88.9)	31 (100)	7 (50)

Abbreviations: IQR, interquartile range; WT, wild-type.

^*a*^ *RET/PTC* Wt, WT, that is, no *RET/PTC.*

^*b*^ *RET/PTC* rearrangement: including *RET* fusions with *CCDC6*, *NCOA4*, *TIMM23B*, *ERC1*, *AKAP13*, *BMS1*, *FKBP15*, *TRIM24*, *RUFY3*, and *SPECC1L*.

^*c*^ *TERTp* Wt, WT, that is, no *TERT* promoter mutation.

^*d*^ *TERT* promoter mutations, including C228T, C250T, and C242T.

**Table 2.** Concurrence test of interactions between *RET/PTC* rearrangements and *TERT* promoter mutations in papillary thyroid cancer

PTC patients	Concurrence index*^a^*	95% CI	*P^b^*
Overall (N = 669)	0.840	0.538-1.314	.427
*BRAF/RAS* Wt*^c^* (N = 163)	2.040	1.110-3.747	.022

Abbreviations: PTC, papillary thyroid cancer; WT, wild-type.

^*a*^Concurrence index = [n(*RET/PTC* Mt, *TERT*p Mt)/n(*TERT*p, Mt)]/[n(*RET/PTC* Mt, *TERT*p Wt)/n(*TERT*p, Wt)].

^*b*^ *P* value for chi-square test.

^*c*^ *BRAF/RAS* Wt, WT, that is, no *BRAF/RAS* mutation.

When dividing the patients with WT *BRAF/RAS* into 4 genotype groups, mortality rates were found to be 5 of 100 (5%) in patients harboring neither mutation, 2 of 18 (11.1%) in patients harboring a *TERT* promoter mutation alone, 0 of 31 (0%) in patients harboring a *RET/PTC* alone, and 7 of 14 (50%) in patients harboring both genetic alterations (Table 3). These results demonstrated a strong association of the genetic duet of coexisting *RET/PTC* rearrangement and *TERT* promoter mutation with the highest mortality. Compared with the group with neither genetic alteration, the *TERT* promoter mutation alone had only a modest effect, *RET/PTC* alone had no effect at all, and coexisting *RET/PTC* and *TERT* promoter mutations showed a robust synergistic effect on mortality. Thus, *RET/PTC* alone has a limited oncogenic function, but it can be converted by the coexisting *TERT* promoter mutation into a strong driver for the aggressiveness of PTC.

**Table 3.** Hazard ratios of effects of various genotypes in papillary thyroid cancer on patient mortality in comparison with the genotype of wild-type *BRAF* and *RAS* and no *RET/PTC* rearrangement in Cox regression model

Genetic alterations			Mortality rate n/N (%)	HRs			
*BRAF* or *RAS* mutation*^a^*	*RET/PTC* rearrangement*^b^*	*TERT*p mutation*^c^*		Univariable model		Multivariable model*^d^*	
				HR (95% CI)	*P*	HR (95% CI)	*P*
−	−	−	5/100 (5.0)	1 (reference)	N/A	1 (reference)	N/A
−	−	+	2/18 (11.1)	2.469 (0.405-14.022)	.308	3.110 (0.332-29.113)	.320
−	+	−	0/31 (0)	3.296e-09 (0-Inf)	.999	3.206e-11 (0-Inf)	.999
−	+	+	7/14 (50)	9.019 (2.635-30.870)	<.001	13.061 (2.338-72.953)	.003
+	−	−	9/293 (3.1)	0.532 (0.179-1.578)	.255	0.465 (0.317-8.641)	.550
+	−	+	37/213 (17.4)	3.073 (1.239-7.621)	.015	2.605 (1.073-6.323)	.034
+*^e^*	+*^e^*	+	44/227 (19.4)	3.375 (1.386-8.220)	.007	2.817 (1.189-6.673)	.019

Abbreviations: HR, hazard ratio; N/A, not available; PTC, papillary thyroid cancer.

^*a*^ *BRAF* or *RAS* alternation, including *BRAF* mutations of *BRAF* V600E and *BRAF* G469A, and *BRAF* fusions with *AKAP9*, *EXOC4*, and *SND1*; and *RAS* mutations of G13R, Q61K, and Q61R in *KRAS*, *HRAS*, and *NRAS* genes.

^*b*^ *RET/PTC* rearrangements, including *RET* fusions with *CCDC6*, *NCOA4*, *TIMM23B*, *ERC1*, *AKAP13*, *BMS1*, *FKBP15*, *TRIM24*, *RUFY3*, and *SPECC1L*.

^*c*^ *TERT* promoter mutations, including C228T, C250T, and C242T.

^*d*^Adjusted for patient race, sex, and age at the diagnosis of PTC.

^*e*^Patients with PTC harboring *BRAF* mutation or *RAS* mutation or *RET/PTC* rearrangement.

### Synergized Effects of *RET/PTC* and *TERT* Promoter Mutations on Poor Patient Survival in Papillary Thyroid Cancer on Kaplan-Meier Analyses

**Figure 1.**
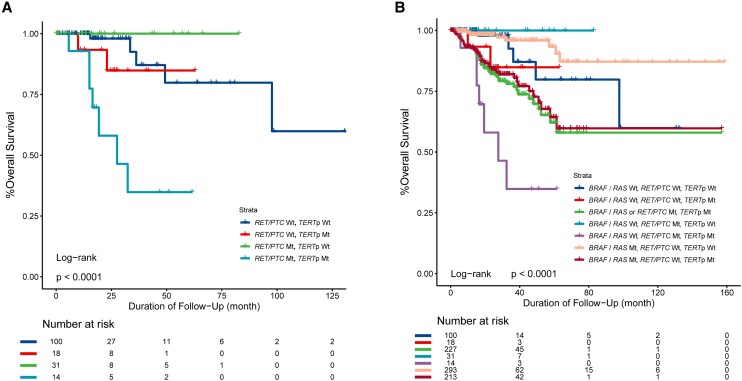
Kaplan-Meier analysis of patient survival curves in papillary thyroid cancer with various genotypes. A, Synergistic effects of *RET/PTC* and *TERT* promoter mutations. A total of 163 patients with wild-type (WT) *BRAF/RAS* pooled from the MSK-MetTropism, MSK-IMPACT, and TCGA-THCA data sets underwent Kaplan-Meier analyses of overall survival in the 4 genotype groups shown. *RET/PTC* Wt-*TERT*p Wt, patients with both WT *RET/PTC* and *TERT* (ie, no *RET/PTC* rearrangements and no *TERT* promoter mutations); *RET/PTC* Wt-*TERT*p Mt, patients with WT *RET/PTC* and mutated *TERT*; *RET/PTC* Mt-*TERT*p Wt, patients with *RET/PTC* rearrangement and WT *TERT*; *RET/PTC* Mt-*TERT*p Mt, patients with both *RET/PTC* rearrangement and mutated *TERT*. B, Synergistic effects of *BRAF/RAS* mutation or *RET/PTC* and *TERT* promoter mutations. A total of 669 patients with complete clinical follow-up and genetic data underwent Kaplan-Meier analyses of overall survival in the 7 genotype groups shown. *BRAF/RAS* Wt-*RET/PTC* Wt-*TERT*p Wt, patients with WT *BRAF* and *RAS*, no *RET/PTC* rearrangement and with WT *TERT*; *BRAF/RAS* Wt-*RET/PTC* Wt-*TERT*p Mt, patients with WT *BRAF* and *RAS*, no *RET/PTC* rearrangement, and with mutated *TERT*; *BRAF/RAS* or *RET/PTC* Mt-*TERT*p Mt, patients with mutated *BRAF* or *RAS,* or *RET/PTC* rearrangement and mutated *TERT*; *BRAF/RAS* Wt-*RET/PTC* Mt-*TERT*p Wt, patients with WT *BRAF* and *RAS*, with *RET/PTC* rearrangement and WT *TERT*; *BRAF/RAS* Wt-*RET/PTC* Mt-*TERT*p Mt, patients with WT *BRAF* and *RAS*, with *RET/PTC* rearrangement and mutated *TERT*; *BRAF/RAS* Mt-*RET/PTC* Wt-*TERT*p Wt, patients with mutated *BRAF* or *RAS*, no *RET/PTC* rearrangement and with WT *TERT*; *BRAF/RAS* Mt-*RET/PTC* Wt-*TERT*p Mt, patients with mutated *BRAF* or *RAS*, no *RET/PTC* rearrangement and with mutated *TERT*.

On the Kaplan-Meier survival analysis of patients with WT *BRAF/RAS*, the survival curve was flat in patients harboring *RET/PTC* alone, slightly declined in patients harboring the *TERT* promoter mutation alone, and sharply declined in patients harboring both genetic alterations (Fig. 1A). Cox regression analysis comparing with patients harboring no genetic alterations (no *BRAF*, *RAS*, *RET/PTC*, or *TERT* promoter mutations) showed survival HRs (95% CI) of 2.469 (0.405-14.022) for *TERT* promoter mutation alone, 3.296e-09 (0-inf) for *RET/PTC* alone, and 9.019 (2.635-30.870) for their coexistence (see Table 3); the former 2 were both insignificant, the latter was robustly significant, and these HRs remained essentially unchanged after adjustment for patient race, sex, and age, demonstrating a strong synergistic effect of the 2 genetic alterations on poor patient survival (see Table 3). To compare the effects of coexisting *RET/PTC* and *TERT* promoter mutations with those of coexisting *BRAF/RAS* mutation and *TERT* promoter mutation on clinical outcomes of PTC, we divided the 669 patients with complete clinical follow-up and genetic data into 7 groups according to the genetic status of *BRAF*, *RAS*, *RET/PTC*, and *TERT* (Fig. 1B). Consistent with previous reports (9, 12), the survival curve of patients with a coexisting *BRAF*/*RAS* mutation and *TERT* promoter mutation declined sharply. The survival curve decline was also sharp when patients with a coexisting *BRAF/RAS* mutation or *RET/PTC* rearrangement and *TERT* promoter mutation were grouped into one entity (Fig. 1B). Cox regression analysis comparing with patients harboring no genetic alterations (no *BRAF*, *RAS*, *RET/PTC*, or *TERT* promoter mutations) showed mortality HRs (95% CI) of 3.073 (1.239-7.621) for a coexisting *BRAF*/*RAS* mutation and *TERT* promoter mutation; and 3.375 (1.386-8.220) for a coexisting *BRAF/RAS* mutation or *RET/PTC* rearrangement and *TERT* promoter mutation (see Table 3).

### Selective Regulation of the Mutated *TERT* but not the Wild-Type *TERT* by RET/PTC

**Figure 2.**
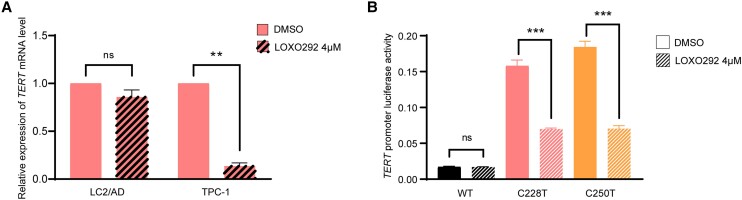
*TERT* promoter mutation-dependent suppression of *TERT* gene expression and *TERT* promoter activities by RET/PTC inhibition. A, *TERT* promoter mutation-dependent suppression of *TERT* gene expression by the RET/PTC inhibitor LOXO292 (selpercatinib). *TERT* messenger RNA (mRNA) expression was not affected by treatment with LOXO292 in LC2/AD cells that harbored *RET/PTC* and wild-type (WT) *TERT*, but was nearly completely suppressed by the inhibitor in TPC-1 cells that harbored both *RET/PTC1* and *TERT* promoter mutation (*TERT* C228T). B, *TERT* promoter mutation-dependent suppression of the *TERT* promoter activities by the RET/PTC inhibitor LOXO292. Luciferase reporter assay was performed using *TERT* promoters carrying various genetic variants as indicated and transfected in TPC-1 cells. Treatment of cells with LOXO292 did not affect the WT *TERT* promoter, but dramatically reduced the activities of *TERT* promoters carrying mutation C228T or C250T. Dimethyl sulfoxide (DMSO) was used as vehicle control. **P less than .01; ***P less than .001. Error bars represent SEM. Experimental details are described in “Materials and Methods.”

Treatment with the RET/PTC inhibitor LOXO292 dramatically suppressed *TERT* gene expression in TPC-1 cells harboring *RET/PTC* and *TERT C228T*, but not in LC2/AD cells harboring *RET/PTC* and WT *TERT* (Fig. 2A). Luciferase report assay using TPC-1 cells demonstrated that mutated *TERT* promoter C228T and C250T activities were significantly suppressed by treatment with the RET/PTC inhibitor LOXO292, while the WT *TERT* promoter activity was not affected (Fig. 2B). Thus, RET/PTC regulated the *TERT* gene in a *TERT* promoter mutation-dependent manner; it robustly upregulated the mutated *TERT*.

### Regulation of the Mutated *TERT* by RET/PTC Through the Mitogen-Activated Protein Kinase Pathway

**Figure 3.**
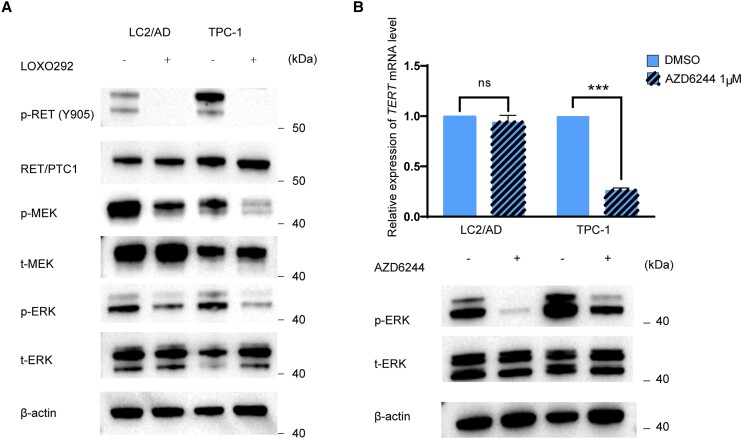
RET/PTC regulates mutant *TERT* through the MAP kinase signaling pathway. A, Suppression of the *MAP* kinase pathway signaling by inhibition of RET/PTC. Shown is the robust suppression by the RET/PTC inhibitor LOXO 292 (selpercatinib) of phosphorylation of RET, MEK, and ERK in the MAP kinase pathway both in LC2/AD and TPC1 cells, which both harbored *RET/PTC*, demonstrating the strong control of the MAP kinase signaling pathway by RET/PTC. B, Suppression of mutated *TERT* expression by blocking the *MAP* kinase pathway. Shown are the lack of suppression of *TERT* messenger RNA (mRNA) expression by the MEK inhibitor AZD6244 in LC2/AD cells, which harbored *RET/PTC* and wild-type (WT) *TERT* and nearly complete suppression of *TERT* mRNA expression by AZD6244 in TPC-1 cells, which harbored both *RET/PTC* and mutated *TERT* promoter. Phosphorylated ERK (p-ERK), total ERK (t-ERK), and β-actin were detected by Western blotting (lower panel). ***P less than .001. Error bars represent SEM. Experimental details are described in “Materials and Methods.”

As shown in Fig. 3A, inhibition of RET/PTC using LOXO292 completely suppressed RET phosphorylation, accompanied by the dramatic reduction of the phosphorylation of MEK and ERK both in TPC-1 and LC2/AD cells, consistent with the known role of RET/PTC in the activation of the MAPK pathway. Similar to the treatment with RET/PTC inhibitor LOXO292 (see Fig. 2A), treatment with the MEK inhibitor AZD6244 also dramatically suppressed *TERT* gene expression in TPC-1 cells harboring *TERT* C228T, but not in LC2/AD cells harboring WT *TERT* (Fig. 3B). Thus, RET/PTC selectively regulated the mutated *TERT* gene through activating the MAPK pathway.

### Conversion of Mutant *TERT* to Wild Type by CRISPR/Cas9 Editing Results in Loss of Effect of *RET/PTC*

**Figure 4.**
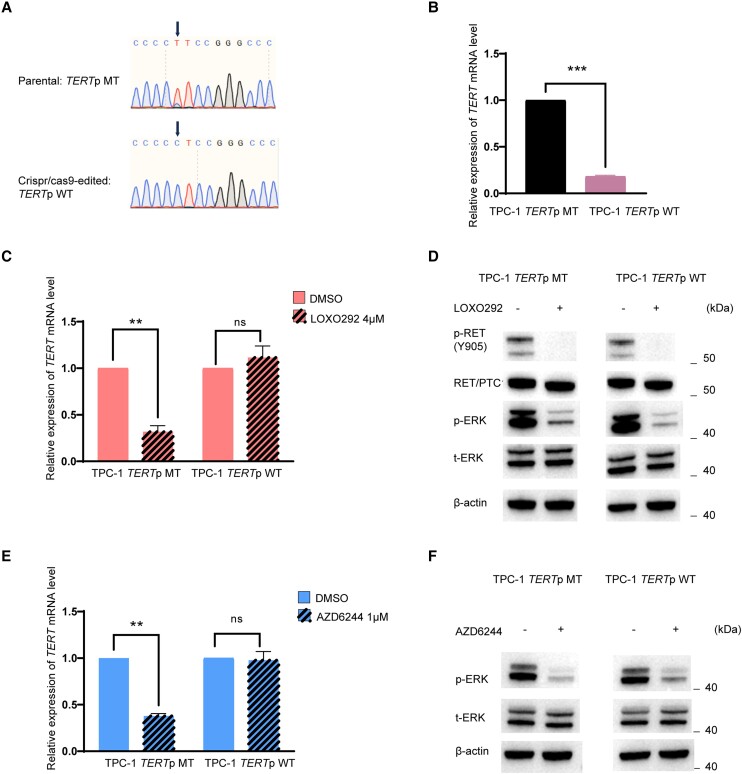
Confirmation of *TERT* promoter mutation-dependence of *RET/PTC* regulation of the *TERT* gene by CRISPR/Cas9 editing. A, Conversion of mutated *TERT* promoter to the wild type (WT). Parental TPC-1 cells naturally harbored *RET/PTC1* and *TERT* C228T, and the latter was converted to WT (T back to C) through CRISPR/Cas9-editing. B, Conversion of mutant *TERT* to WT reduced *TERT* gene expression. Compared with parental TPC-1 cells, *TERT* gene expression was dramatically downregulated in CRISPR/Cas9-edited TPC-1 cells. C, *TERT* promoter mutation-dependent suppression of the *TERT* gene expression by *RET/PTC* inhibition. *TERT* gene expression was suppressed dramatically in parental TPC-1 cells but not in CRISPR/Cas9-edited TPC-1 cells by the RET/PTC inhibitor LOXO292 (selpercatinib). D, Suppression of the *RET/PTC/MAP* kinase pathway signaling by *RET/PTC* inhibition. Phosphorylation of RET and ERK in the MAP kinase pathway was nearly completely inhibited by the RET/PTC inhibitor LOXO292 (selpercatinib) both in parental TPC-1 cells and CRISPR/Cas9-edited TPC-1 cells. E, *TERT* promoter mutation-dependent suppression of *TERT* gene expression by *MAP* kinase pathway inhibition. *TERT* gene expression was suppressed dramatically in parental TPC-1 cells but not in CRISPR/Cas9-edited TPC-1 cells by the MEK inhibitor AZD6244 (Selumetinib). F, Suppression of the *MAP* kinase pathway signaling by *MEK* inhibition. Phosphorylation of ERK in the MAP kinase pathway was nearly completely inhibited by the MEK inhibitor AZD6244 (selumetinib) both in parental TPC-1 cells and CRISPR/Cas9-edited TPC-1 cells. **P less than .01; ***P less than .001. Error bars represent SEM. Experimental details are described in “Materials and Methods.”

Conversion of *TERT C228T* to WT by CRISPR/Cas9 editing in TPC-1 cells (Fig. 4A) induced a dramatic downregulation of *TERT* gene expression (Fig. 4B). The RET/PTC inhibitor LOXO292 dramatically suppressed *TERT* expression in parental TPC-1 cells harboring *RET/PTC* and *TERT C228T* but had no effect on CRISPR/Cas9-edited TPC-1 cells harboring *RET/PTC* and WT *TERT* (Fig. 4C), while RET/PTC and MAPK pathway were inhibited in both cells (Fig. 4D). Similarly, the MEK inhibitor AZD6244 dramatically suppressed *TERT* expression in parental TPC-1 cells but not in CRISPR/Cas9-edited TPC-1 cells (Fig. 4E), although it inhibited the MAP kinase pathway in both cells (Fig. 4F). These results demonstrated again the *TERT* promoter mutation-dependence of the action of *RET/PTC*.

### *RET/PTC* Regulates Mutant *TERT* Through Mitogen-Activated Protein Kinase Pathway Involving *GABPB*

**Figure 5.**
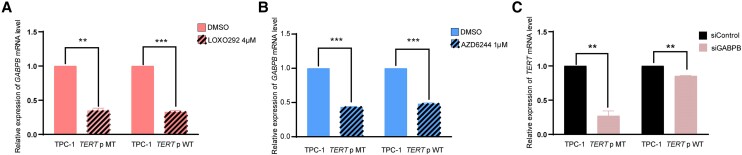
Involvement of GABPB in the *RET/PTC* regulation of mutant *TERT* by the MAP kinase pathway. A, Suppression of *GABPB* gene expression by *RET/PTC* inhibition. *GABPB* expression was dramatically suppressed by the RET/PTC inhibitor LOXO292 both in parental TPC-1 cells harboring *RET/PTC* and *TERT* C228T and CRISPR/Cas9-edited TPC-1 cells harboring *RET/PTC* and wild-type (WT) *TERT*. B, Suppression of *GABPB* gene expression by *MAP* kinase pathway inhibition. *GABPB* expression was dramatically suppressed by the MEK inhibitor AZD6244 (selumetinib) both in parental TPC-1 cells and CRISPR/Cas9-edited TPC-1 cells. C, Preferential suppression of the expression of mutant *TERT* over WT *TERT* by *GABPB* knockdown. *TERT* messenger RNA (mRNA) level was suppressed dramatically in parental TPC-1 cells but only minimally in CRISPR/Cas9-edited TPC-1 cells by GABPB knockdown. **P less than .01; ***P less than .001. Eerror bars represent SEM. Experimental details are described in “Materials and Methods.”

Since the BRAF V600E/MAP kinase pathway regulated mutant *TERT* through regulating *GABPB* (22-27), we investigated whether this was also the case with RET/PTC. Treatment with the RET/PTC inhibitor LOXO292 dramatically suppressed the *GABPB* gene expression both in parental TPC-1 cells and CRISPR/Cas9-edited cells (Fig. 5A). Similarly, treatment with the MEK inhibitor AZD6244 also dramatically suppressed the *GABPB* gene expression both in parental TPC-1 cells and CRISPR/Cas9-edited cells (Fig. 5B). In contrast, knockdown of *GABPB* resulted in suppression of *TERT* gene expression dramatically in parental TPC-1 cells but only minimally in CRISPR/Cas9-edited TPC-1 cells (Fig. 5C). Thus, RET/PTC selectively regulated the mutant *TERT* through the MAP kinase pathway involving GABPB as BRAF V600E did (22-27).

### PI3K/AKT Pathway Is not Involved in the Regulation of Mutant *TERT* by RET/PTC

**Figure 6.**
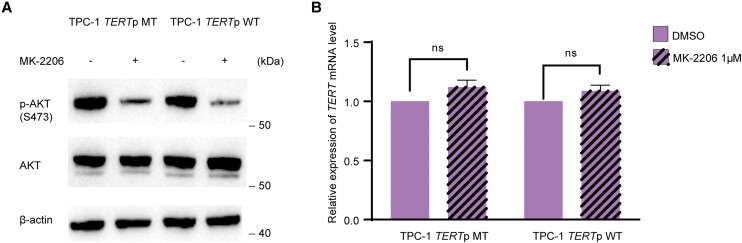
The PI3K/AKT pathway is not involved in the regulation of *TERT* expression by RET/PTC. A, Suppression of the PI3K/AKT pathway by treatment with AKT inhibitor. Phosphorylation of AKT in the PI3K pathway was nearly completely inhibited by the AKT inhibitor MK-2206 both in parental TPC-1 cells and CRISPR/Cas9-edited TPC-1 cells. B, Suppression of the PI3K pathway did not affect *TERT* expression. *TERT* gene expression was not suppressed in parental TPC-1 cells and CRISPR/Cas9-edited TPC-1 cells by treatment with the AKT inhibitor.

Considering that RET/PTC can activate both MAPK and PI3K/AKT pathways (35), we investigated whether the PI3K/AKT pathway was also involved in the regulation of mutant *TERT* expression by RET/PTC. Treatment with the protein kinase B (AKT) inhibitor MK-2206 inhibited the PI3K/AKT pathway both in parental TPC-1 cells (harboring *RET/PTC* and *TERT C228T*) and CRISPR/Cas9-edited TPC-1 cells (harboring *RET/PTC* and WT *TERT*) (Fig. 6A) but did not affect *TERT* expression in either cell (Fig. 6B). These results suggest that the regulation of mutant *TERT* by RET/PTC does not involve the PI3K/AKT pathway.

## Discussion

*RET/PTC* rearrangements are among the most common genetic alterations in the MAPK pathway in PTC (4, 5, 28-30), but, unlike *BRAF/RAS* mutations, their role in the clinical outcomes of PTC is controversial and remains to be clearly undefined. Numerous studies have demonstrated a robust oncogenic function of the genetic duets of *BRAF/RAS* mutations and *TERT* promoter mutations in driving tumor aggressiveness and poor clinical outcomes of PTC (8-18). The underlying molecular mechanism is that the *BRAF/RAS* mutation-activated MAPK pathway robustly and selectively upregulates the mutated *TERT* gene, but not the WT *TERT* (22-27). TERT, at aberrantly elevated levels, is a strong oncoprotein that promotes tumorigenesis and cancer aggressiveness involving its noncanonical telomerase-independent functions, including, prominently, the suppression of apoptosis (41). In these studies on the role of *BRAF/RAS* mutations and *TERT* promoter mutations, *RET/PTC* rearrangements have not been considered. Given that *BRAF/RAS* mutations and *RET/PTC* rearrangements are mutually exclusive among themselves in the MAPK pathway and they each can independently activate the pathway, we speculated that *RET/PTC*, when coexisting with *TERT* promoter mutations, could, like *BRAF/RAS* mutations, also selectively upregulate the mutant *TERT* through activating the MAPK pathway, thus promoting tumor aggressiveness and poor clinical outcomes of PTC.

Indeed, the present study proved this hypothesis to be true. Specifically, we demonstrated that, like the *BRAF* mutation, *RAS* mutation, and *TERT* prompter mutation, each alone had little effect in previous studies, the *RET/PTC* and *TERT* promoter mutation each alone also had little effect in the present study; in fact, *RET/PTC* alone had no effect at all on patient survival. In striking contrast, a robust synergistic effect between *RET/PTC* and *TERT* promoter mutation on the mortality of PTC patients was observed. A previous study demonstrated that GABP in the form of complex GABPA and GABPB could directly bind the mutant *TERT* promoter to activate and upregulate the *TERT* gene (7). Our group demonstrated that the BRAF V600E-activated MAPK pathway upregulated the mutant *TERT* through upregulating the *GABPB* gene and promoting the formation of the GABP complex (23). In the present study, we demonstrated that RET/PTC used a similar mechanism to regulate mutant *TERT*. Specifically, our in vitro studies showed that the action of *RET/PTC* in the genetic duet with *TERT* promoter mutations, like that of *BRAF/RAS* mutations in their genetic duet with *TERT* promoter mutations, was mediated through activating the MAPK pathway and *GABPB*, leading to the selective activation of the mutated *TERT*, but not the WT *TERT*. Thus, the normally ineffective *RET/PTC* can be functionally converted by *TERT* promoter mutations into a strong oncogenic genetic driver promoting the aggressiveness of PTC. Based on these results, it is reasonable to propose that PTC harboring only *RET/PTC* can be treated in a relatively conservative manner while PTC harboring coexisting *RET/PTC* and *TERT* promoter mutations should be treated in a relatively aggressive manner. The present study may explain the previous findings of mild oncogenic behaviors of *RET/PTC* in general but aggressive behaviors in a portion of cases, as *RET/PTC* alone is much more common than its coexistence with *TERT* promoter mutations as shown in the present study (Supplementary Fig. S1 in the supplementary materials (39)). The present study is also consistent with the long-time knowledge that pediatric PTC has a far better prognosis than adult PTC even though *RET/PTC* is far more common in the former than the latter (4, 5, 42), probably because *TERT* promoter mutations are very uncommon, hence with very rare coexistence of *RET/PTC* and *TERT* promoter mutations, in the former (42). The present study also demonstrated that the genetic duet of *RET/PTC* and *TERT* promoter mutations had similar effects to those of the genetic duet of *BRAF*/*RAS* and *TERT* promoter mutations on clinical outcomes of PTC. Therefore, PTCs with a coexisting *BRAF*/*RAS* mutation or *RET/PTC* rearrangement and *TERT* promoter mutation could be clinically considered as one disease entity with common poor clinical outcomes.

The RET/PTC inhibitor LOXO292 (selpercatinib) has been approved by the US Food and Drug Administration to treat *RET*-altered cancers, including advanced thyroid cancer harboring *RET/PTC*, with good therapeutic responses in some patients but not in others (43). The strong inhibitory effects of LOXO292 on RET, the MAPK pathway, and the mutated *TERT* gene, but not the WT *TERT* gene in cells harboring *RET/PTC* observed in the present study, suggest that it is possible that the patients that responded well to LOXO292 harbored the genetic duet of *RET/PTC* and *TERT* promoter mutations while those that did not respond well harbored only *RET/PTC*. In a recent in vitro and animal study we demonstrated that BRAF/MEK inhibitors could induce strong therapeutic responses in cancer cells harboring both *BRAF* V600E and mutant *TERT* promoter through causing robust apoptosis but not cells harboring WT *TERT* (24). A subsequent German clinical trial on patients with melanoma demonstrated that BRAF/MEK inhibitors could induce much superior therapeutic responses in patients harboring a coexisting *BRAF* mutation and *TERT* promoter mutation compared with patients harboring only a *BRAF* mutation (25). We proposed that *BRAF* mutation-induced activation of the MAPK pathway and hence elevated TERT in mutated *TERT* cancers, which is a strong suppressor of apoptosis, made such cancer cells evolutionarily become dependent on this molecular signaling system for powerful survival (through apoptosis resistance); once this system was disrupted, as achieved by the inhibition of the MAPK pathway, expression of the mutated *TERT* gene suddenly shut off, removing the suppression of apoptosis and resulting in robust cancer cell apoptosis (24). We speculate that this is the same mechanism that the genetic duet of *RET/PTC* and mutated *TERT* may confer on PTC’s particularly good responsiveness to the treatment with RET/PTC inhibitors. In this context and given that a MEK inhibitor could strongly suppress *TERT* in *RET/PTC*-harboring cancer cells in the present study, it is possible that MEK inhibitors, like RET/PTC inhibitors, may also be therapeutically effective in PTC harboring the genetic duet of *RET/PTC* and *TERT* promoter mutations.

A limitation of the present study was the relatively small number of cases with the genetic duet of *RET/PTC* and *TERT* promoter mutations, being 8.59% in patients with PTC harboring the WT *BRAF/RAS*. However, the robust role of this genetic duet demonstrated in the present study suggests that PTC with it is a distinct clinical entity, making its identification important for genetic-guided precision management. Given the large patient population with PTC globally, a considerable number of patients are expected to harbor the genetic duet of *RET/PTC* and *TERT* promoter mutations and thus potentially benefit from treatment with MEK inhibitors. *RET* fusion genes are also seen in other human cancers, such as non–small cell lung cancer (44). The findings in PTC in the present study may likely be applicable also to the management of such cancers. Another limitation of this study is the limited availability of *RET/PTC*-positive cell lines, which are rare in the field. Nevertheless, the 2 precious cell lines of this type used in the present study showed robust results, strongly supporting our conclusions. Moreover, this limitation was mitigated by CRISPR/Cas9 editing to generate a new genotype of the TPC-1 cell that was equivalent to a new *RET/PTC* cell line.

In summary, we for the first time report a robust, synergistic oncogenic role of the genetic duet of coexisting *RET/PTC* and *TERT* promoter mutations with no role of the *RET/PTC* alone in poor clinical outcomes of PTC. Like the genetic duet of *BRAF/RAS* mutations and *TERT* promoter mutations, the genetic duet of *RET/PTC* and *TERT* promoter mutation also identifies PTC with high mortality, making this PTC clinically unique. As such, PTCs harboring coexisting a *BRAF*/*RAS* mutation or *RET/PTC* rearrangement and *TERT* promoter mutation can be clinically considered as one disease entity sharing a common poor prognosis. This study provides new implications for genetic-based prognostication as well as the potential of effective therapeutic targeting of RET and MEK guided by *RET*/*PTC* and *TERT* status in PTC patients. It is therefore advisable that genetic patterns of *RET/PTC* and *TERT* promoter be added to the current genetic-based risk stratification and precision management of PTC.

## Data Availability

Original data generated and analyzed during this study are included in this published article or in the data repositories listed in “References.”

## Abbreviations

AKT: protein kinase B
HR: hazard ratio
IQR: interquartile range
MAPK: mitogen-activated protein kinase kinase
PTC: papillary thyroid cancer
*TERT*: telomerase reverse transcriptase
WT: wild-type
